# *Phenethyl isothiocyanate* Triggers Apoptosis, Combats Oxidative Stress and Inhibits Growth of Ehrlich Ascites Carcinoma Mouse Model

**Published:** 2018

**Authors:** Nada H. Eisa, Heba S. Said, Nehal M. ElSherbiny, Laila A. Eissa, Mamdouh M. El-Shishtawy

**Affiliations:** a *Department of Biochemistry, Faculty of Pharmacy, Mansoura University, Mansoura 35516, Egypt. *; b *Department of Microbiology, Faculty of Pharmacy, Mansoura University, Mansoura 35516, Egypt.*

**Keywords:** Apoptosis, Bax, Bcl-2, Caspase-9, Ehrlich ascites carcinoma, Phenethyl isothiocyanate

## Abstract

The aim of this study is to investigate the antitumor activity and possible molecular mechanism of *Phenethyl isothiocyanate* (*PEITC*) against Ehrlich ascites carcinoma *in-vivo* and *in-vitro*.* In-vivo*, ascetic fluid volume, body weight, serum malondialdehyde (MDA) level and total antioxidant capacity (TAC) were determined using Ehrlich ascites carcinoma (EAC) bearing mice. *In-vitro,* MTT assay was used. RT-PCR was used to investigate role of *PEITC* in apoptosis by analyzing the expression of Bax, caspase-9, and Bcl-2 genes. The effect of *PEITC* on caspase-9 enzyme activity was also tested. *PEITC* and/or Doxorubicin (Dox) treatment significantly suppressed EAC growth as compared to EAC/oil control mice. *PEITC* treatment showed a dose-dependent inhibition of EAC cells as indicated by MTT assay. We found that significant increase in MDA level and decrease in TAC caused by Dox treatment were significantly reduced by combination with *PEITC* treatment. Bax, caspase-9 genes’ expression and caspase-9 enzymatic activity were significantly increased, while Bcl-2 gene expression was significantly decreased in *PEITC* treated mice. *PEITC* may act as a promising anticancer agent either alone or more effectively in combination with Dox through apoptotic cell death induction.

## Introduction

Cancer is considered as the second cause of mortality around the world where up to 12.7 million patients have been diagnosed with cancer and this number is expected to reach 21 million within the next 15 years (1, 2). 

Currently used conventional chemotherapeutic agents have limited safety, efficacy, and showed resistance upon long term use (3). Doxorubicin (Dox) is an anthracycline antibiotic that is used for treatment of solid and hematopoietic tumors (4, 5). Dox treatment causes tumor cell death through intercalation into DNA base pairs in addition to generation of reactive oxygen species (ROS) (6). However, Dox treatment is restricted due to its severe adverse effects and multidrug resistance (7).

Discovery of new, effective, and safe anti-tumor agents, either from natural or synthetic sources, is still a challenge for many biochemical researchers. Several natural products have been studied for their anti-tumor effect and as a matter of fact more than 50% of available anti-tumor agents have been obtained from natural sources (8).

**Figure 1:**
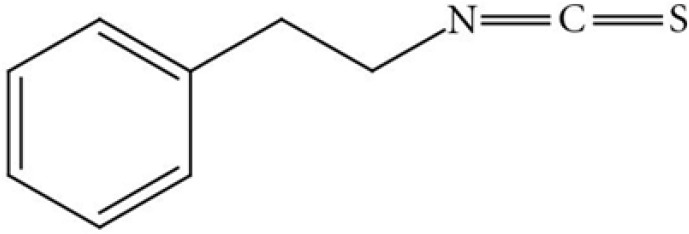
Chemical structure of *Phenethyl* *isothiocyanate* (*PEITC*). IUPAC (2-Isothiocyanatoethyl) benzene

Cruciferous vegetables such as watercress and broccoli that are rich in isothiocyanates (ITCs) have been reported to reduce the incidence of different types of cancers (9, 10). *Phenethyl isothiocyanate* (*PEITC*) (Figure 1) is an important member of ITC family that has been extensively studied for its chemo-preventive action (11). *PEITC* exerts its anticancer effect through different mechanisms, most importantly, induction of apoptosis (12).

Apoptosis is a self-cellular suicidal mechanism that is controlled by diverse signaling pathways and used to destroy damaged cells (13). Many genes regulate apoptosis, most prominently, the Bcl-2 family that is classified into anti-apoptotic genes such as (Bcl-2 and Bcl-XL) and apoptotic genes such as (Bax and Bak) (14, 15). Bcl-2 and Bax genes work with each other to achieve apoptosis (15). It has been reported that overexpression of Bax, but not Bcl-2, promotes cytochrome c release from the mitochondria which leads to caspase-9 activation that results in initiating a downstream caspase cascade that eventually causes cell death (16).

Accordingly, the present work was designed to evaluate the antitumor and cytotoxic effect of *PEITC* and/or Dox against Ehrlich ascites carcinoma bearing-mice *in-vivo* and on Ehrlich ascites carcinoma cells *in-vitro*. Furthermore, we studied the effect of *PEITC* and/or Dox on expression of some apoptotic genes in order to elucidate the underlying molecular mechanism of *PEITC* action.

## Experimental

*Drugs and Chemicals*

*PEITC*, >98% purity was purchased from Abcam (ab142645, Cambridge, UK). Doxorubicin HCl (Adricin) was purchased from EIMC United Pharmaceuticals (Cairo, Egypt). 3-(4,5-dimethyl-2-thiazolyl) 2,5diphenyl-2H-tetrazolium-bromide (MTT) was purchased from SERVA electrophoresis GmbH (Heidelberg, Germany). Trypan Blue and Dimethyl sulfoxide (DMSO) were purchased from Sigma–Aldrich (St. Louis, MO, USA). Roswell Park Memorial Institute (RPMI) - 1640 medium was purchased from Gibco Laboratories (Life Technologies Inc., Grand Island, NY, USA). Fetal bovine serum (FBS) and penicillin-streptomycin antibiotic were purchased from Lonza (Basel, Switzerland). All other chemicals are of high purity and analytical grade.

*Animals*

Adult Swiss female albino mice (25–30 g) were purchased from Theodore Bilharz Institute, Giza, Egypt and kept in animal house of Faculty of Pharmacy, Mansoura University, Egypt. Animals were kept in standard size polycarbonate cages under standard laboratory conditions (26 ± 1 °C, 12-h light:12-h dark cycle) and had free access to food and water *ad libitum*. Ehrlich ascites carcinoma (EAC) induction started 10 days following animals’ accommodation under these standard conditions. All animal experiments carried out in this study were complied with ″Research Ethics Committee″ Faculty of Pharmacy, Mansoura University, Mansoura, Egypt, in accordance with ″Principles of Laboratory Animal Care″ (NIH publication No. 85-23, revised 1985).

*Ehrlich ascites carcinoma cells*

EAC cell line was obtained from the National Cancer Institute, Cairo University, Cairo, Egypt. EAC cells were maintained by intraperitoneal (IP) passage of 1 X 10^6^ cells in adult female Swiss albino mice (17). Seven days later, the ascetic fluid was collected from mice peritoneal cavity under sterile condition by needle aspiration. Viable tumor cells were counted in a Neubauer hemocytometer using the trypan blue dye (0.4%) exclusion method. EAC cells were used for both *in-vivo* and *in-vitro* experiments.

*Ehrlich ascites carcinoma model*

On day zero, mice were injected intraperitoneally (IP) with 1 X 10^6^ EAC cells (0.1 mL/mouse) for tumor induction. On the next day, mice were weighted then randomly divided into 4 groups; each group consists of 10 animals. Treatments were administered for 14 days as follows: 

EAC/oil control group that received 0.1 mL oil/mouse/day orally, *PEITC*-treated group that received *PEITC* at a dose of 60 mg/kg/0.1 mL oil/mouse/day orally (18), Dox-treated group that received IP injection of Dox at a dose of 2 mg/kg/0.1 mL/mouse/day (19) and *PEITC*/Dox combination treated group that received Dox (2 mg/kg/0.1 mL/mouse/ day, IP) 2 h following receiving *PEITC* (60 mg/kg/0.1 mL oil/mouse/day, orally). A fifth group of eight mice that received 0.1 mL oil/day/mouse orally was used as normal control group. On day 15, animals were weighted, blood was collected by retro-orbital puncture and then sacrificed. Serum samples were prepared by centrifugation of blood samples that was withdrawn via retro-orbital puncture at 4000 rpm for 10 min at 4 °C. Ascetic fluid was aspirated from all animals for measuring tumor volume as an indication for the antitumor effect. Ascetic fluid samples were centrifuged at 1800 rpm for 5 min at 4 °C. The pellet was flash-frozen in liquid nitrogen then stored in -80 °C till used.

*Malondialdehyde and total antioxidant capacity assay*

Commercially available malondialdehyde (MDA) and total antioxidant capacity (TAC) colorimetric assay kits from Biodiagnostic Company (Giza, Egypt) were used for spectrophotometric determination of serum MDA levels and serum TAC according to the methods reported by Satoh (20) and Koracevic *et al*. (21), respectively.

*Cell culture*

EAC cells were cultured in RPMI-1640 medium supplied with 10% FBS and 1% (v/v) penicillin-streptomycin antibiotic. They were maintained at 37 °C in a humidified atmosphere with 5% CO_2_ (Binder, C-series, Germany).

*Cell viability assay*

MTT assay was used for the assessment of cell viability. EAC cells (1 X 10^5^ cells/well) were inoculated in 96-well flat bottom tissue culture plate (Griener, Germany). One day after, cells were treated with three different concentrations of *PEITC* (5, 10 and 20 µmol/L), Dox (100 µmol/L), a combination of *PEITC* 20 µmol/L + Dox 100 µmol/L and DMSO with a final concentration of 0.1% that have been used as a control. Cells were incubated for 24 h and incubation period continued for additional 4 h at 37 °C following the addition of 20 μL/well of MTT solution (5 mg/mL in PBS). The formazan crystals formed as a result of MTT reduction were solubilized by treating cells with 100 µL of 0.04 N acidic isopropanol. Absorbance was measured at 540 nm using a microplate reader, (Bio-Tek ELX800, USA). The experiment was repeated three times. Viable cells were calculated as percentage relative to DMSO treated control cells.

*Quantitative, Real-Time Polymerase Chain Reaction (RT-PCR) for Bax, caspase-9 and Bcl-2 gene expression*

Total RNA was isolated from EAC cells using TRIzol^®^ Reagent (Ambion, Life Technologies, USA) according to the manufacturer’s instructions. The quantity and quality of the isolated RNA was assessed spectrophotometrically at 260 nm and 260/280 nm ratio, respectively, using NanoPhotometer^®^ (Implen, GmbH, Germany). One microgram of the total RNA was reverse transcribed into single-stranded complementary DNA using QuantiTect Reverse Transcription Kit (Qiagen, USA) according to the manufacturer’s instructions. The mRNA expression level of apoptosis genes Bax, caspase-9 and Bcl-2 was determined in different EAC cells. For normalization of gene expression, mouse Glyceraldehyde 3-phosphate dehydrogenase (GAPDH) was quantified in parallel with target genes. Reactions were performed using HOT FIREPol EvaGreen qPCR Mix (Solis BioDyne, Tartu, Estonia) in Rotor-Gene Q (Qiagen, USA). Gene specific primers are summarized in Table 1. The primers were designed using PREMIER Biosoft (USA) according to gene sequence obtained from PubMed (Entrez Gene), blasted on NCBI/Blast and purchased from Invitrogen-Life Technologies. Thermal cycling program was as follows: initial activation cycle at 95 °C for 15 min followed by 40 cycles at 95 °C for 15 sec for denaturation, 65 °C for 20 sec for annealing and finally 72 °C for 20 sec for elongation. Relative expression of studied genes was determined using 2^−ΔΔCT^ method relative to GAPDH. The specificity of the designed primers and the size of amplified PCR products were confirmed by melt curve analysis and 2% agarose gel electrophoresis, respectively.

**Table 1:** Primers used for amplification of studied genes in qRT-PCR

**Gene of Interest**	**Primer Sequence**	**Reference Sequence**	**Product size (bp)**
GAPDH Forward	5`-ATGGTGAAGGTCGGTGTGAAC-3`	NM_008084.3 NM_001289726.1	251
GAPDH Reverse	5`-TTGATGTTAGTGGGGTCTCGC-3`		
Bax Forward	5`-CCACCAGCTCTGAACAGATC-3`	NM_007527.3	140
Bax Reverse	5`-CAGCTTCTTGGTGGACGCAT-3`		
Caspase-9 Forward	5`-TGGACATTGGTTCTGGCG-3`	NM_015733.5 NM_001277932.1	117
Caspase-9 Reverse	5`-TGTTGATGATGAGGCAGTGG-3`		
Bcl-2 Forward	5`-GGATGACTTCTCTCGTCGCTAC-3`	NM_009741.4 NM_177410.2	199
Bcl-2 Reverse	5`-TGACATCTCCCTGTTGACGCT-3`		

*Caspase-9 enzyme activity colorimetric assay*

EAC cells collected from control and treated groups were lysed using chilled lysis buffer provided by Caspase-9 Colorimetric Assay Kit (BioVision, CA, USA) and then caspase-9 enzyme activity was determined spectrophotometrically according to the manufacturer’s instructions.

*Statistical analysis*

Results are presented as mean ± standard error of mean (SEM). One-way analysis of variance (ANOVA) followed by Tukey’s post-hoc test using GraphPad Prism 6.01 (GraphPad Software, SanDiego, CA, USA) was used to find out statistically significant results. *P*-value of less than 0.05 was considered statistically significant.

## Results

*Effect of PEITC on EAC cell growth in-vivo*

**Figure 2:**
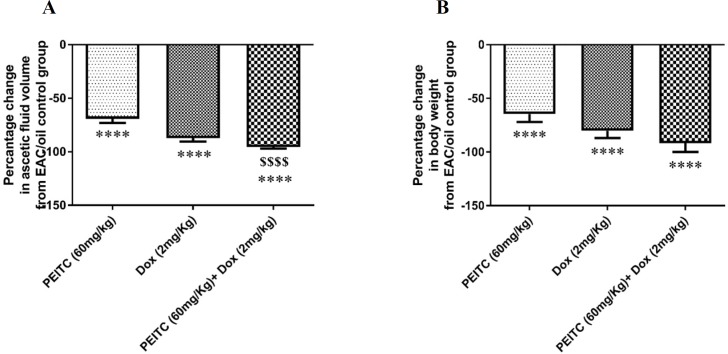
Effect of *PEITC* and/or Dox treatments on (A) ascetic fluid volume and (B) body weight change. ^****^Significant as compared with the EAC/oil group at *P* < 0.0001. ^$$$$^Significant as compared with the *PEITC *group at *P *< 0.0001

*PEITC* treatment inhibited EAC cell growth as indicated by the decrease in ascetic fluid volume and the change in body weight. *PEITC* treatment and Dox treatment showed a significant decrease in ascetic fluid volume by (69.14% and 87.37%), respectively as compared to EAC/oil control group. Furthermore, *PEITC*/Dox combination therapy significantly decreased ascetic fluid volume by (95.42%) as compared to EAC/oil control group (Figure 2A). Body weight of all animals at day (15) was measured and body weight change from the initial body weight was calculated. *PEITC*, Dox and *PEITC*/Dox treated groups showed a significant decrease in body weight change as compared to EAC/oil control group by (64.5%, 80.13% and 91.64%), respectively (Figure 2B).

*Effect of PEITC on EAC cell growth in-vitro*

MTT assay was used for testing EAC cell viability. As compared to DMSO treated EAC control cells, *PEITC* treatment (5, 10, and 20 µmol/L) for 24 h showed a significant dose-dependent decrease in EAC cell viability by 22.59%, 33.06% and 42.39%, respectively. Dox treatment (100 µmol/L) for 24 h significantly decreased cell viability by 53.41%. Combination of *PEITC* (20 µmol/L) and Dox (100 µmol/L) treatments for 24 h caused a significant decrease in cell viability by 75.29% 

(Figure 3).

**Figure 3:**
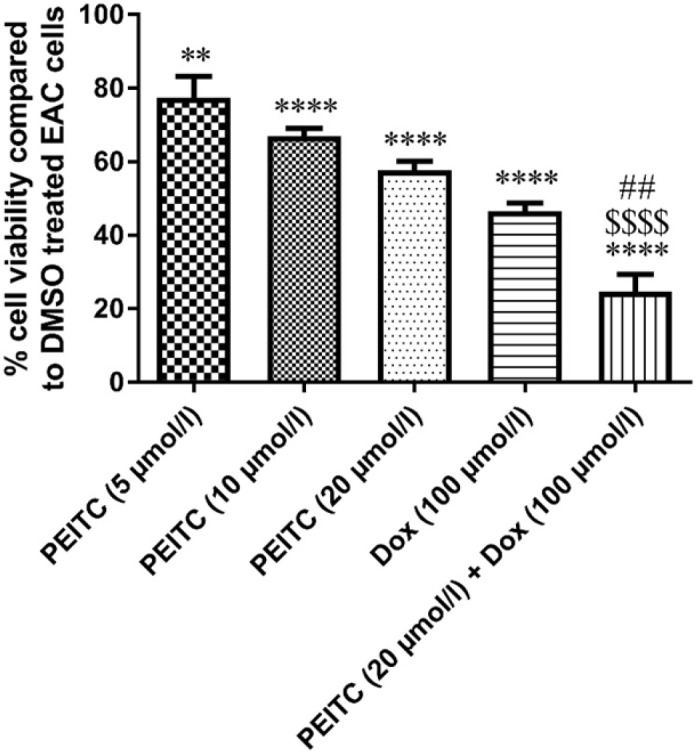
Effect of *PEITC* and/or Dox treatments on EAC cell viability (*in-vitro*). ^****^Significant as compared with the control group at *P* < 0.0001. ^**^Significant as compared with the control group at *P* < 0.01. ^##^Significant as compared with the Dox group at *P* < 0.01. ^$$$^Significant as compared with the *PEITC* (20 µmol/L) group at *P* < 0.0001

*Effect of PEITC on malondialdehyde level and total antioxidant capacity*

**Figure 4:**
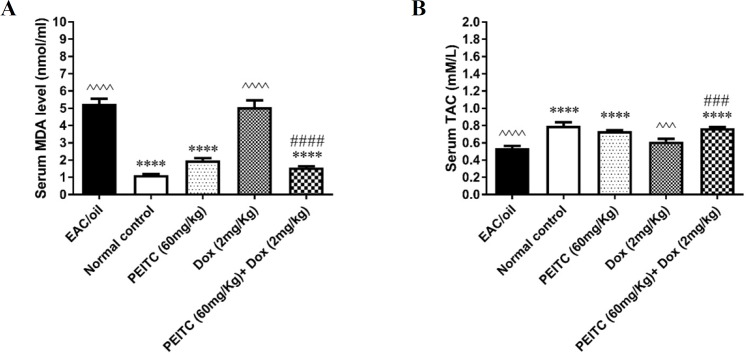
Effect of *PEITC* and/or Dox treatments on (A) serum MDA level and (B) serum TAC

As shown in Figures 4A and 4B, serum MDA level of EAC/oil control group and mice receiving Dox treatment significantly increased as compared to normal control group (*P <* 0.0001 and *P <* 0.0001, respectively). On the contrary, serum TAC of EAC/oil control group and mice receiving Dox treatment significantly decreased as compared to normal control group (*P <* 0.0001 and *P =* 0.0001, respectively). *PEITC* treatment significantly decreased serum MDA level and increased serum TAC as compared to EAC/oil control group (*P <* 0.0001 and *P <* 0.0001, respectively). *PEITC*/Dox combination treatment significantly decreased serum MDA level as compared to EAC/oil control group (*P <* 0.0001) and Dox treated group (*P <* 0.0001). On the other hand, *PEITC*/Dox combination treatment significantly increased serum TAC as compared to EAC/oil control group (*P <* 0.0001) and Dox treated group (*P =* 0.0006).

*Effect of PEITC on caspase-9 enzymatic activity*

**Figure 5:**
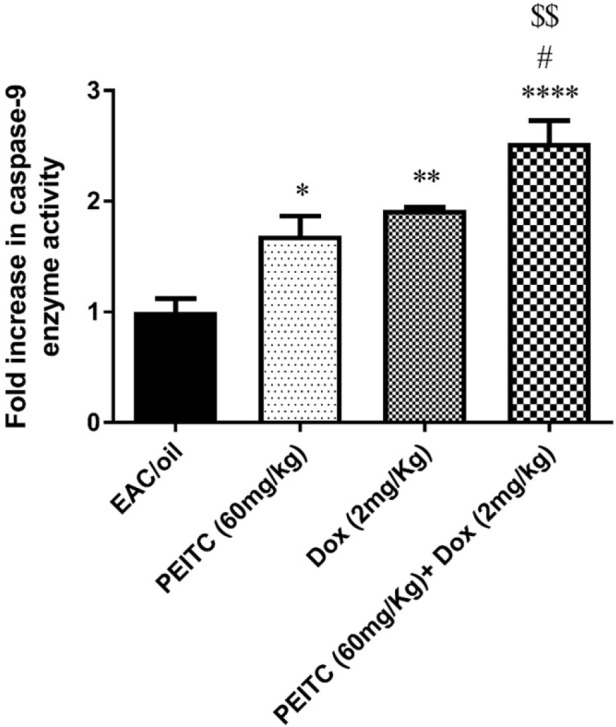
Effect of *PEITC* and/or Dox treatments on caspase-9 enzyme activity. ^****^Significant as compared with the EAC/oil group at *P* < 0.0001. ^**^Significant as compared with the EAC/oil group at *P* < 0.01. ^*^Significant as compared with the EAC/oil group at *P* < 0.05. ^#^Significant as compared with the Dox group at *P* < 0.05. ^$$^Significant as compared with the *PEITC* group at *P* < 0.01

As shown in Figure 5, *PEITC*, Dox and *PEITC*/Dox treated groups significantly enhanced caspase-9 enzymatic activity as compared to EAC/oil control group by 1.69 (*P =* 0.02), 1.92 (*P =* 0.002) and 2.52 (*P <* 0.0001) fold, respectively. Moreover, *PEITC*/Dox combination therapy significantly increased caspase-9 enzymatic activity as compared to both *PEITC* treated group (*P =* 0.004) and Dox treated group (*P =* 0.04).

*Effect of PEITC on Bax, caspase-9 and Bcl-2 gene expression*

**Figure 6:**
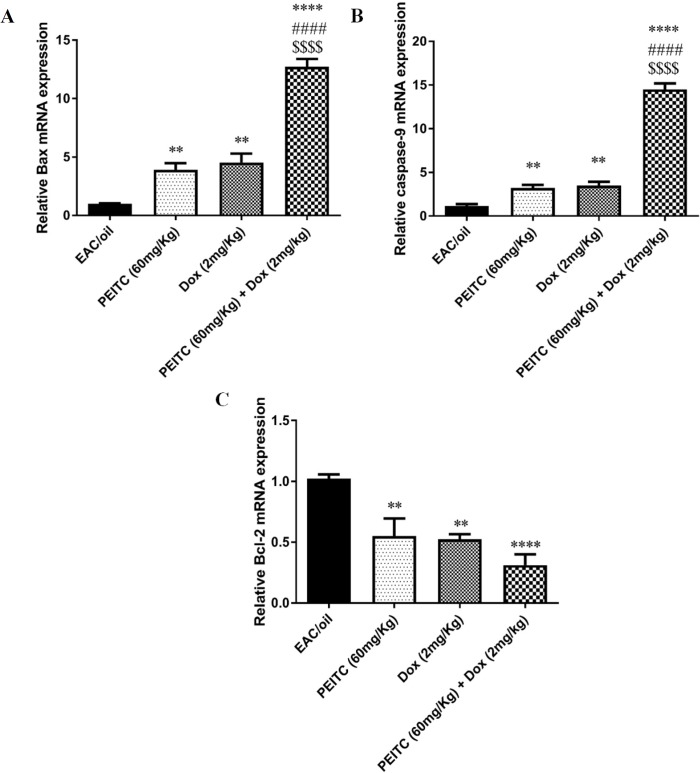
Effect of *PEITC* and/or Dox treatments on (A) Bax, (B) caspase-9 and (C) Bcl-2 genes’ expression. ^****^Significant as compared with the EAC/oil group at *P* < 0.0001. ^**^Significant as compared with the EAC/oil group at *P* < 0.01. ^####^Significant as compared with the Dox group at *P* < 0.0001. ^$$$$^Significant as compared with the *PEITC* group at *P* < 0.0001

Our results have shown that *PEITC* treatment significantly increased expression level of both Bax and caspase-9 genes as compared to their levels in EAC/oil control group by 3.92 (*P* = 0.006) and 3.2 (*P* = 0.009) fold, respectively. Similarly, Dox treatment significantly increased expression level of both genes by 4.53 (*P* = 0.001) and 3.49 (*P* = 0.002) fold, respectively. *PEITC*/Dox combination treatment significantly increased expression level of both genes by 12.71 (*P* < 0.0001) and 14.5 (*P* < 0.0001) fold, respectively in comparison with their levels in EAC/oil control group. Moreover, combination therapy significantly increased expression level of Bax and caspase-9 genes, as compared to their levels in *PEITC* treated group (*P* < 0.0001 and *P* < 0.0001) and Dox treated group (*P* < 0.0001 and *P* < 0.0001), respectively (Figures 6A and 6B).

On the contrary, *PEITC*, Dox and *PEITC*/Dox treatments significantly decreased expression level of Bcl-2 gene as compared to its level in EAC/oil control group by 0.55 (*P =* 0.004), 0.52 (*P =* 0.002) and 0.31 (*P* < 0.0001) fold, respectively. *PEITC*/Dox combination treatment decreased Bcl-2 expression level as compared to its level in both *PEITC* treated group and Dox treated group, though that decrease was not statistically significant (Figure 6C).

## Discussion

*PEITC* is a promising anti-cancer agent that showed inhibitory effect against tumor growth in different previous studies (11, 22 and 23). Although apoptosis induction is considered the main mechanism for *PEITC* anti-tumor action, the exact molecular mechanism for apoptosis induction has not been fully investigated (24). In this study, the possible underlying molecular mechanism behind anti-tumor and cytotoxic effects of *PEITC* compared to and in combination with Dox were investigated using Ehrlich ascites carcinoma model *in-vivo* and *in-vitro*.

Our results from *in-vivo* study showed that either *PEITC* treatment or Dox treatment solely caused a significant inhibition of EAC cell growth as indicated by the significant decrease in ascetic fluid volume and body weight of treated mice as compared to EAC/oil group. Upon combination of both *PEITC* and Dox treatments, further decrease in ascetic fluid volume was observed as compared to *PEITC* treatment alone. These results were further supported by *in-vitro* study, where *PEITC* treatment showed inhibition of EAC cellular viability in a dose-dependent manner. Interestingly, the cytotoxic effect of highest dose of *PEITC* treatment when combined with Dox treatment significantly exceeded the cytotoxic effect of each treatment alone. Altogether, our results showed that *PEITC* and/or Dox treatments effectively inhibit EAC cellular growth *in-vivo* and *in-vitro*.

One of the possible mechanisms by which *PEITC* exerts its antitumor activity, is the induction of apoptosis (25). It has been reported that *PEITC* was able to induce apoptosis in different types of cultured human cancer cell lines (26-28). Apoptosis is a well-regulated pathway by which multicellular organisms get rid of unwanted damaged cells (29). It usually ends up with activation of caspase cascade via either intrinsic or extrinsic pathway (30). The Bcl-2 family genes regulate apoptosis as it has been reported that their expression levels are altered in response to anticancer agents (31, 32).

In agreement with previous studies (33-36), our results showed that *PEITC* treatment increased the expression level of both Bax and caspase-9 genes and decreased the expression level of Bcl-2 gene as compared to EAC/oil control group. These results were further reinforced by the ability of *PEITC* treatment to enhance caspase-9 enzymatic activity. Interestingly, *PEITC*/Dox combination treatment surpassed each treatment alone in increasing the expression level of Bax and caspase-9 genes and in enhancing caspase-9 enzymatic activity but not in decreasing Bcl-2 gene expression level.

Alteration of measured pro/anti-apoptotic genes in EAC cells upon *PEITC* and/or Dox treatment may confirm induction of apoptotic pathway. This could be further explained by the release of cytochrome c from mitochondria as a result of increase in Bax and decrease in Bcl-2 expression. Cytochrome c forms an apoptosome with apoptotic protease activating factor 1 and pro-caspase-9, consequently caspase-9 activation, pro-caspase-3 induction and eventually apoptosis (37, 38).

Carcinogenesis is a multistep process that is initiated and promoted by multiple factors; most prominently oxidative stress (39). Free radicals and lipid peroxidation which ends up with MDA production mediate oxidative stress in different cells and tissues (40, 41). Their role in tumor progression has been widely studied (42, 43). Free radicals and MDA cause extensive tissue and cellular damage, especially affecting lipoprotein part of cell membrane (44). Besides *PEITC* antitumor activity, it has been reported that *PEITC* exerts antioxidant activity (45). This agreed with our results which showed that mice treated with *PEITC* caused a significant decrease in MDA level and significant increase in TAC as compared to EAC/oil control group. 

On the contrary, MDA level in mice receiving Dox treatment was significantly increased unlike TAC, which was significantly decreased as compared to normal control group. This was in agreement with Lorenzo *et al.* (46), who reported that Dox induces production of free radicals and lipid peroxidation. In addition, Mukherjee *et al.* (47) found that Dox could significantly decrease the activity of some antioxidant enzymes such as superoxide dismutase and glutathione peroxidase. When *PEITC* treatment was combined with Dox treatment, MDA level was significantly decreased and TAC was significantly increased in comparison with Dox treatment alone. Collectively, *PEITC*/Dox combination treatment showed a significant anti-tumor effect without causing significant oxidative damage. This could be explained by the ability of *PEITC* to induce some antioxidant enzymes whose activity was reduced by Dox treatment (48, 49), thus antioxidant enzymes could capture free radicals (50).

Taken together, our results indicated that *PEITC* efficaciously suppressed EAC tumor growth *in-vivo* and *in-vitro *via alteration of apoptotic genes and its marked antioxidant effect. It can be concluded that *PEITC* is useful as natural therapy that could be considered for cancer treatment along with Dox. However, further research is required to fully establish the antitumor effect of *PEITC*/Dox combination before considered for clinical trial.
